# Comparative study on salivary IL- 8 and LDH among tobacco users without lesions, leukoplakia, oral submucous fibrosis and oral squamous cell carcinoma

**DOI:** 10.6026/973206300213542

**Published:** 2025-10-31

**Authors:** Srinivasan Venkatesh, Saraswathi Gopal K, Nandhini Ganesan

**Affiliations:** 1 Department of Oral medicine and Radiology, Meenakshi Ammal Dental College and Hospital, MAHER University, Chennai, India

**Keywords:** Interleukin-8, Lactate dehydrogenase (LDH), oral squamous cell carcinoma, saliva, ELISA

## Abstract

Oral and oropharyngeal malignancies, OSCC accounts for 95% of cases. Diagnosis and prognosis of OSCC relies upon the tumour staging, an important predictor of survival. Therefore, it is of interest to evaluate the changes in the salivary levels of IL-8 and LDH among Tobacco users without lesions, Individuals with Leukoplakia, Oral submucous fibrosis and OSCC. 40 cases were analysed in this study. We show that salivary IL8 and LDH which can serve as one reliable biomarker in early detection of OSCC and a prognostic indicator for OPMDs.

## Background:

OSCC contributes for 95% of all oral and oropharyngeal cancers [1]. Oral cancer is one vital global health concern, ranking as 6th most frequent cancer globally, primarily driven by carcinogen exposure. India experiences highest incidence of oral cancer, largely due to widespread use of tobacco [2]. OPMDs (Oral Potentially Malignant Disorders) are present clinically with an elevated risk of progressing to OSCC. The most common OPMDs comprise OSMF (oral submucous fibrosis), oral Leukoplakia, Oral Lichen planus, erythroleukoplakia [3]. Early detection significantly enhances survival rates, making the timely diagnosis of potentially malignant disorders crucial in reducing incidence of OSCC [4]. Biopsy stays gold standard for diagnosing oral cancer [5]. Several tumor markers have been identified in serum, oral tissues, and saliva, which aid in early diagnosis and serve as potential prognostic indicators in patients with oral squamous cell carcinoma [6]. Interleukin (IL)-8 is an 8 kD protein, initially isolated from LPS (lipopolysaccharide)-stimulated peripheral blood mononuclear cells [7]. It has also been found that a range of tumors produce IL-8 constitutively and in response to cytokines [8]. At normal to supranormal levels, SCC cells form wide range of potent cytokines. Among those, one immunomodulatory cytokines, IL-8, is renowned physio pathological factor with chemotactic as well as angiogenic functions [7, 9]. Fresh culture cell lines, surgical specimens and distinct cultured cell lines of head and neck SCC all contain IL-8 [10]. LDH is one such enzyme presents in saliva. Although this enzyme is found in almost every tissue, it is most prevalent in the heart, liver, kidneys, red blood cells, muscles, brain and lungs. Various studies demonstrated salivary LDH expression in patients with OSCC and oral potentially malignant disorder and found significantly higher in the patient group as opposed to control group [11]. Therefore, it is of interest to evaluate salivary level of Interleukin - 8 and LDH in patients with Leukoplakia, OSMF, OSCC and tobacco users without lesions.

## Materials and Methods:

This prospective, randomized cross-sectional study involved a total of 40 participants selected from Outpatient Department of Oral Medicine and Radiology at Meenakshi Ammal Dental College and Hospital, Chennai. The study population was divided into four distinct sub-groups. Total 40 cases have been included in ongoing investigation and evenly categorized into four groups, with 10 subjects in every group. Group I consisted of tobacco users without any clinically evident lesions, having a minimum history of three years of tobacco use. Group II included patients clinically diagnosed with leukoplakia, while Group III comprised patients clinically diagnosed with OSMF. Group IV included patients who were clinically diagnosed and histopathologically verified to have OSCC. Patients having any history of allergies, infectious diseases or acute/subacute dental abscess, pregnant and lactating women, patients with underlying systemic illnesses and presently gone through or have undergone any type of definitive therapy for OSCC in form of radiation, chemotherapy or any other additional treatments were excluded. Saliva samples were collected under non-stimulatory condition. 3-5ml of salivary samples was collected before any therapeutic procedures which have been kept at -4°C. To eliminate debris and squamous cells, sample has been transferred to 15ml threaded centrifuge tube and spun at 2500 RPM for 15 minutes for Interleukin 8 estimation whereas 1000 revolutions per minute (RPM) for 10 minutes for LDH estimation. In order to estimate IL-8, resulting supernatant has been separated, aliquoted and kept in a freezer at -90°C for further ELISA examination. In accordance with manufacturer's suggested protocols, LDH has been evaluated utilizing LDH enzyme kit using standard biochemical method with an ultraviolet-visible spectrophotometer.

## Results:

Statistical data analysis has been done utilizing IBM SPSS Statistics for Windows, Version 26.0. Armonk, NY: IBM Corp. Descriptive statistics have been measured for various clinical parameters, including mean, range, minimum value, maximum value, and standard deviation. Normality of data assessed utilizing Shapiro-Wilk test revealed that data have been distributed normally. Therefore, further analysis was done using parametric tests like ANOVA and the post hoc Tukey test. Significance level in present research has been kept at p<0.05. In Table 1 (see PDF), Salivary IL8 shows overall statistically highly significant difference between the study groups (Group I to Group IV) with a P-value of 0.00 (P<0.01). In Table 2 (see PDF) and Figure 1 (see PDF), Salivary IL-8 values were higher in Group 2, 3, 4 than 1. A highly significant difference was also observed when comparing the OSCC group with Leukoplakia and OSMF groups (P < 0.001). However, we did not find any significant difference in IL8 values when comparing Group 2 (Leukoplakia) and Group 3 (OSMF), which might be due to the similar potentially malignant lesions. Table 3 (see PDF) Salivary LDH shows overall statistically highly significant difference among research groups (Group I to IV). In Table 4 (see PDF) Salivary LDH values were higher in the Leukoplakia group (P < 0.05), OSMF group (P < 0.001) and OSCC group (P < 0.001) compared to the Group 1 (Controls), and this difference was significant statistically. A highly significant difference was also observed when comparing the OSCC group with Leukoplakia and OSMF group (Figure 2 - see PDF). It is therefore inferred that both salivary IL-8 and LDH values progressively increase towards the change to carcinoma/ malignant condition than in tobacco users without any lesions.

## Discussion:

Oral cancer is a broad terminology that includes various malignant lesions that present in the oral tissues. More than 90% of all oral neoplasms are thought to be OSCCs [12]. 2-4% of all cancer cases worldwide are oral cancers [13, 14]. Although the etiopathogenesis of OSCC is complex, tobacco and tobacco related products are important contributors. Oral cancer may be preceded by potentially malignant disorders, which should alarm the person to be cautious over his/her habits and make a changeover of their lifestyle [15]. During ongoing investigation, we detected significant rise in IL-8 and LDH level in OSCC in comparison with OSMF, Oral Leukoplakia as well as tobacco users without lesions. In 2019, Rezaei *et al.* [16] performed a meta-analysis to compare salivary along with serum levels of IL-6 and IL-8 in patients with OSCC to controls. After reviewing 309 studies from four databases, 26 studies were selected for analysis. Serum and salivary levels of IL-6 and IL-8 have been shown to be considerably greater in OSCC patients than in controls that have been following our research, suggesting that these cytokines may be useful biomarkers for OSCC early identification. In 2021, Elena Ferrari *et al.* [17] conducted a systematic review on salivary cytokines as biomarkers for OSCC. Total 27 observational studies were included based on the established inclusion and exclusion criteria. Among cytokines most commonly examined as potential OSCC biomarkers, IL-6, IL-8, and TNF-α be available at higher concentrations in saliva of OSCC patients contrary to healthy controls according to our research. These cytokines may therefore act as foundation for developing rapid tests for early diagnosis of oral cancer. In 2016, Lokesh *et al.* [18] performed one research to assess precision of salivary LDH as possible biomarker for OSCC diagnosis as well as for establishing a correlation among salivary LDH levels and the tumor's histological differentiation. A control group of 20 patients and 30 patients with clinical and histological diagnoses of OSCC have been selected for research. In conclusion, patients with OSCC had considerably higher LDH readings which were in correlation with current research. In 2022 Bhuvaneshwari *et al.* [19] LDH levels in saliva of patients with oral cancer, leukoplakia, smokers without lesions, and controls were estimated and compared within the groups. LDH values displayed marked elevation in leukoplakia group and OSCC group when compared to controls and smokers, as per our research. In current research, mean LDH values in OSCC group have been determined to be higher than in OSMF group, Leukoplakia and tobacco users. Most of investigations in several populations exposed favourable results which disclosed effectiveness of salivary Interleukin - 8 and LDH in OSCC and OPMDs.

## Conclusion:

New paradigms are arising and making the medical specialities enthroned in pivoting carcinomatous conditions. Serum biomarkers have become reliable investigative modality for decades. During current years, literature preserves utilization of salivary biomarkers as reliable detective source in field of cancer. We show that salivary IL-8 and LDH which can serve as one reliable biomarker in early detection of OSCC and a prognostic indicator for OPMDs.

## Future perspectives:

Larger Samples are needed to standardize salivary IL-8 and LDH as predictable biomarker as it would aid in more expected outcomes.

## Patient consent:

Obtained

## Source of financial support:

Nil

## References

[1] Skrinjar I (2015). Med Oral Patol Oral Cir Bucal..

[2] Piyarathne NS (2021). Expert Rev. Mol..

[3] Selvam NP, Sadaksharam J (2015). Asia-Pac J Clin Oncol..

[4] Sahibzada HA (2017). Diagnostics..

[5] Chaurasia A (2021). Natl J Maxillofac Surg..

[6] Cottin SC (2018). Cancers of the Head & Neck..

[7] Oppenheim JJ (1991). Annu Rev Immunol..

[8] Abruzzo LV (1992). Am J Pathol..

[9] Moore BB (1999). Am J Pathol..

[10] Richards BL (1997). Am J Surg..

[11] Goyal G (2020). APJCP..

[12] Choi S, Myers JN (2008). J Dent Res..

[13] Williams HK (2000). Mol Pathol..

[14] Nocini R (2020). Ann Cancer Epidemiol..

[15] Jiang X (2019). Tob Induc Dis..

[16] Rezaei F (2019). J. Interferon Res..

[17] Ferrari E (2021). Int. J. Mol. Sci..

[18] Lokesh K (2016). J Clin Diagn Res.

[19] Bhuvaneswari M (2022). J. Cancer Res. Ther..

